# Ethical and Methodological Considerations Interviewing Dementia Caregivers

**DOI:** 10.1093/geroni/igab046.1531

**Published:** 2021-12-17

**Authors:** Quinton Cotton, Laura Block, Clark Benson, Amanda Friz, Britta Chelgren, Brady Stroik, Emily Ploch, Andrea Gilmore Bykovskyi

**Affiliations:** 1 University of Wisconsin - Medicine & Public Health, Fitchburg, Wisconsin, United States; 2 University of Wisconsin-Madison, Madison, Wisconsin, United States; 3 University of Washington, University of Washington, Washington, United States; 4 University of Wisconsin-Madison, University of Wisconsin-Madison, Wisconsin, United States

## Abstract

Greater inclusion of people living with dementia (PLWD) and their caregivers in research is a global research priority and an expressed priority of dementia advocacy organizations. Absent inclusion of PLWD and caregivers, our understanding of dementia-related experiences and optimization of care and caregiving interventions is stymied. Qualitative interviewing techniques constitutes a primary method for obtaining PLWD and caregivers’ perspectives. Yet, there is little guidance on use of qualitative interviewing techniques among PLWD and caregivers or discussion of potential challenges encountered, despite unique vulnerabilities faced throughout the research process, which may be further heightened among historically excluded groups. Meaningful progress toward inclusion of PLWD and their caregivers in dementia research necessitates broader examination of associated methodological and ethical considerations that arise in the conduct of interviews. Drawing from a large multi-site qualitative study of dementia caregivers with exposure to high levels of social disadvantage, we used a multiple-triangulation qualitative approach across interview transcripts, memos, and interviewer discussions to identify methodological and ethical challenges that arose during the interviewing process. Challenges were identified across all phases of research, and included relational concerns with PLWD and family members due to disclosure of sensitive information, risk of re-traumatization in discussing past experiences, multiple roles of caregivers with conflicting perspectives, variable recall capacity, limited prior appraisal of caregiving, and request of interviewers for medical advice or selecting services . We outline events evidencing these challenges and proposed strategies (i.e. use of research consults, interview debriefing) to strengthen research capacity to anticipate and respond to them.

